# Aberrant Nodal Topological Properties and Functional Connectivity of Amygdala Subregions Underlie Emotion–Visceral Integration Impairment in IBS With Depressive Symptoms

**DOI:** 10.1155/np/7203336

**Published:** 2026-07-20

**Authors:** Yidan Liang, Rongting Hou, Liqiang Wu, Yihan Jin, Ruoyu Tang, Yun Guo, Xiaofei Chen, Jie Li

**Affiliations:** ^1^ School of Clinical Medicine, The Affiliated Hospital of Hangzhou Normal University, Hangzhou Normal University, Hangzhou, China, hznu.edu.cn; ^2^ Department of Radiology, The Affiliated Hospital of Hangzhou Normal University, Hangzhou, China, hznu.edu.cn; ^3^ Department of Gastroenterology, The Affiliated Hospital of Hangzhou Normal University, Hangzhou, China, hznu.edu.cn; ^4^ Centre for Cognition and Brain Disorders, The Affiliated Hospital of Hangzhou Normal University, Hangzhou, China, hznu.edu.cn

**Keywords:** amygdala subregion, depression, functional connectivity, graph theory, irritable bowel syndrome

## Abstract

**Background:**

Irritable bowel syndrome (IBS) is a common disorder of brain–gut interaction frequently co‐occurs with depressive symptoms (dIBS). The amygdala is a critical hub for emotion–visceral integration and exhibits functional heterogeneity across its subregions. However, subregion‐specific network alterations in dIBS remain unclear.

**Methods:**

Forty‐nine IBS patients and 36 demographically matched healthy controls (HCs) completed resting‐state functional magnetic resonance imaging (rs‐fMRI) and clinical assessments. Patients were stratified into dIBS (*n* = 28) and nondepressive IBS (ndIBS, *n* = 21) groups. Bilateral lateral amygdala (lAmyg) and medial amygdala (mAmyg) were selected as seeds for graph‐theoretical nodal metrics and seed‐based functional connectivity (FC) analyses. One‐way analysis of covariance with post hoc comparisons was performed to assess intergroup differences. Correlation, mediation, and receiver operating characteristic (ROC) analyses were further conducted.

**Results:**

Compared with ndIBS patients, dIBS patients exhibited increased degree centrality (DC) and nodal efficiency (Ne) in the left lAmyg, which were positively associated with depressive symptom severity. In addition, dIBS patients showed widespread hypoconnectivity between amygdala subregions and prefrontal and sensorimotor regions, including the medial superior frontal gyrus, postcentral gyrus, and thalamus. These FC alterations were correlated with clinical symptoms severity. Notably, the mAmyg‐related FC in superior frontal gyrus and thalamus showed mediation effects linking gastrointestinal symptoms and depressive symptoms and exhibited high accuracy in distinguishing dIBS from ndIBS (area under the curves, AUCs > 0.8).

**Conclusion:**

Aberrant nodal properties and disrupted connectivity of amygdala subregions may reflect altered emotion–visceral integration in dIBS. These findings provide neuroimaging evidence for brain–gut interaction abnormalities in dIBS and suggest that amygdala subregional networks may serve as potential markers for symptom stratification and targeted interventions.

## 1. Introduction

Functional gastrointestinal disorders (FGIDs) are characterized by disrupted brain–gut interactions [1]. As one of the most common FGIDs, irritable bowel syndrome (IBS) affects ~5%–10% of the global population [2]. Its primary symptoms encompass chronic abdominal pain and disturbed bowel habits, with no detectable structural abnormalities [2]. Psychiatric comorbidities, particularly depression, affect nearly 30% of IBS patients [3], contributing to a bidirectional cycle that exacerbates both gastrointestinal and affective symptoms. IBS with comorbid depressive symptoms (dIBS) is associated with poorer quality of life and increased healthcare utilization, representing a major public health issue [4, 5].

IBS pathogenesis involves visceral hypersensitivity and altered central processing [1]. Neurobiological models suggest that visceral pain is mediated by distributed brain networks, in which the thalamus and insula encode sensory‐discriminative features, while the prefrontal cortex contributes to the cognitive dimension [6]. Beyond these sensory‐modulatory dimensions, increasing evidence highlights the amygdala as a central hub for integrating visceral input with emotional processing [6–8]. Our prior research [9] demonstrated that dIBS patients exhibit more prominent dysregulation within the emotional arousal network. Consistent with this, Quidé et al. [10] have reported altered amygdala‐prefrontal connectivity in chronic pain with comorbid depression, suggesting enhanced negative emotional processing. Experimental evidence further indicates that modulation of amygdala‐related circuits can influence both visceral hypersensitivity and depression‐like behaviors [11]. Notably, abnormalities in emotion–arousal regions appear to diminish after controlling for negative affect in IBS patients, suggesting that depressive comorbidity contributes to distinct neural mechanisms [12]. Together, these findings underscore the importance of stratified investigation based on the affective status.

Crucially, the amygdala comprises functionally heterogeneous subregions [13, 14] with distinct roles in emotion–pain integration and brain–gut regulation. The lateral amygdala (lAmyg) primarily integrates sensory and visceral inputs from the thalamus and sensory cortices [15], whereas the medial amygdala complex (mAmyg) modulates autonomic and stress‐related responses via projections to the hypothalamus and brainstem [16, 17]. Alterations in these subregions have been implicated in the emotional evaluation of visceral perceptions [18]. Importantly, such functional heterogeneity suggests that examining the amygdala as a single structure may obscure subregion‐specific alterations relevant to symptom heterogeneity in IBS. However, whether these amygdala subregions exhibit distinct alterations in network topology and FC in relation to depressive comorbidity in IBS remains unclear.

To address this gap, the present study combined graph‐theoretical analysis and seed‐based functional connectivity (FC) analysis using resting‐state functional magnetic resonance imaging (rs‐fMRI) to characterize both global network properties (e.g., degree centrality [DC], nodal efficiency [Ne]) and subregion‐specific connectivity patterns of the amygdala. Using the Brainnetome 246 Atlas, the present study compared these neural measures among dIBS patients, nondepressive IBS (ndIBS), and healthy controls (HCs). Furthermore, correlation, mediation, and receiver operating characteristic (ROC) analyses were performed to examine associations with clinical symptoms and their value in distinguishing depressive comorbidity status. By integrating subregion‐specific topology, connectivity, and clinical associations, this study provides a refined characterization of amygdala‐related alterations in dIBS and highlights their potential relevance for stratified clinical assessment.

## 2. Materials and Methodology

### 2.1. Participants

Our team recruited 49 patients diagnosed with IBS and 36 demographically matched HCs for sex, age, and educational level from the hospital’s health center and via public advertisements. This cohort was previously described in our work [9], with the current work extends this research by investigating the topological attributes of subregion in the emotion arousal network and the features of whole‐brain FC as the key focus. Signed informed consent was voluntarily provided by all subjects before their enrollment.

Patients with IBS were clinically diagnosed in accordance with the Rome III diagnostic criteria, and all subjects underwent detailed medical history orientation, physical examination, and colonoscopy. Participants were required to be right‐handed and aged between 18 and 65 years. We excluded IBS patients who met any of the following criteria: a current or past history of major neuropsychiatric disorders; a history of cerebral injury, brain surgery, or other significant neurological conditions; use of antidepressants, probiotics, or prokinetic agents within 2 weeks prior to enrollment; substance abuse; intellectual impairment; and any contraindications to MRI scanning. Correspondingly, exclusion criteria for HCs were defined as follows: any current or past medical history of gastrointestinal disorders, chronic pain, or significant systemic diseases; neuropsychiatric or significant neurological disorders; substance or alcohol abuse; and any contraindications to MRI scanning.

The evaluation of depressive symptom severity utilizing the 17‐item Hamilton depression rating scale (HAMD‐17) [19]. Participants with a HAMD‐17 scores >7 were classified as having comorbid depressive symptoms (dIBS group, *n* = 28; HAMD‐17 score [mean ± SD]: 12.75 ± 1.71), while those with a score ≤7 comprised the ndIBS group (*n* = 21; HAMD‐17 score [mean ± SD]: 3.71 ± 1.38).

### 2.2. Multidimensional Scale Assessment

The HAMD‐17 and the 14‐item Hamilton Anxiety Rating Scale (HAMA‐14) [20] was utilized to evaluate the severity of depressive and anxiety symptoms, respectively. With the 15‐item gastrointestinal symptom rating scale (GSRS) [21], gastrointestinal symptom severity was evaluated. In addition to the total GSRS score, subscale scores (abdominal pain, dyspepsia, diarrhea, reflux, and constipation) were also calculated, as recommended in previous studies [22]. Additionally, the duration of gastrointestinal symptoms was recorded for all IBS patients. Following completion of all multidimensional scale assessments, the patients immediately underwent MRI scanning.

### 2.3. MRI Image Acquisition

All imaging data were obtained on a 3.0 T GE Discovery MR‐750 scanner. The applied scanning protocol included high‐resolution three‐dimensional T1‐weighted imaging (3D‐T1) and rs‐fMRI. Routine clinical diagnostic sequences were additionally acquired to screen for structural abnormalities (e.g., mass lesions, cerebral infarcts, and prominent white matter lesions). Participants were requested to maintain motionless and eye closure throughout scanning. Ear protection and foam padding were supplied to dampen scanner‐induced noise and mitigate involuntary head motion. The basic imaging parameters are presented in Table 1.

**Table 1:** MRI scanning parameters.

MRI sequence	Sequence	Repetition time (ms)	Echo time (ms)	Field of view (mm^2^)	Matrix	Voxel size (mm^3^)	Number of slices	Flip angle (°)
3D‐T1	Spoiled gradient echo sequence	8.16	3.18	256 × 256	256 × 256	1 × 1 × 1	176	8
Rs‐fMRI	Gradient echo planar imaging	2000	30	192 × 192	64 × 64	3 × 3 × 3	43	90

*Note:* 3D‐T1, high‐resolution three‐dimensional T1‐weighted imaging.

Abbreviation: Rs‐fMRI, resting‐state functional MRI.

### 2.4. MRI Data Processing

Data processing was performed using the Data Processing Assistant for Resting‐State Functional MR Imaging toolkit (DPARSF, http://rfmri.org/dpabi) [23] and the Graph Theoretical Network Analysis toolkit (GRETNA, http://www.nitrc.org/projects/gretna/), both implemented within the Statistical Parametric Mapping (SPM12; available at www.fil.ion.ucl.ac.uk/spm) software platform.

### 2.5. Data Preprocessing

Preprocessing steps included: (1) transformation of raw DICOM images to NIFTI format and exclusion of the first 10 time points; (2) correction of slice timing and head motion. Data were excluded if translation exceeded 3 mm or rotation exceeded 3° or mean framewise displacement (FD) exceeded 0.2 mm; (3) scrubbing to minimize the influence of minor head movements; (4) tissue segmentation, initial spatial normalization, and nonlinear normalization using the DARTEL algorithm; (5) spatial smoothing with a Gaussian kernel (FWHM = 4 mm) and temporal band‐pass filtering (0.01–0.08 Hz); (6) nuisance signal regression, including linear detrending, the Friston 24‐parameter model for head motion artifacts, and signals from cerebrospinal fluid, white matter, and global signal; and (7) final quality control of all preprocessed data, including visual inspection of normalization accuracy and assessment of FD‐based motion artifacts to ensure no significant intergroup differences in head motion.

### 2.6. Topological Analysis of Amygdala Subregions

A region‐based FC network was constructed using GRETNA. The Brainnetome 246 Atlas was employed to define the nodes of brain networks. It categorizes the whole brain into a total of 246 subregions, of which 210 are cortical and 36 are subcortical [14]. Extraction of the mean time series was carried out for each node. Pearson correlation coefficients between all node pairs were calculated to generate a 246 × 246 FC matrix for each participant. Individual matrices were thresholded and binarized across a sparsity range of 0.05–0.50 (in steps of 0.05) [24]. For each network, the following nodal topological metrics were computed: betweenness centrality (BC) and DC for centrality; Ne and nodal shortest path length (NSp) for long‐range efficiency; and nodal clustering coefficient (NCp) and nodal local efficiency (NLe) for local efficiency. All correlation matrices were Fisher’s *r*‐to‐z transformed prior to group‐level analysis to improve normality. Finally, the aforementioned metrics were extracted from the bilateral lAmyg and mAmyg (regions 211–214) in each individual’s map for subsequent statistical analyses.

### 2.7. Seed‐Based Whole Brain FC Analysis

Based on the preprocessed data, seed‐based whole‐brain voxel‐wise FC analysis was performed using the DPARSF. The bilateral lAmyg and mAmyg (regions 211–214 in the Brainnetome Atlas) served as regions of interests (ROIs). A voxel‐wise analytical approach was subsequently applied to compute Pearson correlation coefficients between the average time series of each ROI and the time series of all voxels throughout the entire brain, thereby generating individual‐level resting‐state FC maps for each amygdala subregion. Individual FC maps were then Fisher’s *r*‐to‐z transformed to satisfy the normality assumption required for subsequent statistical analyses.

### 2.8. Statistical Analysis

Statistical analyses of demographic and clinical data were performed with SPSS Statistics 27.0 software (IBM Corp., Chicago, IL, USA). One‐way analysis of variance (ANOVA) with subsequent post hoc *t*‐tests was applied to evaluate variations in age, years of education, HAMD‐17 scores, and HAMA‐14 scores among the three groups (dIBS, ndIBS, and HCs). Sex distribution was compared using chi‐square tests. Disease duration, total GSRS score, and its subscale scores were compared between the two patient groups using two‐sample *t*‐tests, with a two‐tailed *p* < 0.05 defined as statistical significance.

Group differences in each nodal topological property were examined using one‐way analysis of covariance (ANCOVA) with age, sex, and education as covariates, followed by post hoc tests when the main effect was significant (*p* < 0.05). The analyses were carried out in SPSS.

Using ANCOVA implemented in DPARSF, group differences in seed‐based FC values of the amygdala subregion were explored, with age, sex, and education incorporated serving as covariates. Significant clusters were identified using Gaussian random field (GRF) correction (voxel‐level *p*  < 0.005, cluster‐level *p*  < 0.05). Subsequently, each significant cluster was individually defined as an ROI to separately extract the mean FC values (Fisher’s *z*‐values) for each subject. Bonferroni‐corrected pairwise comparisons were then performed between each pair of the groups within each ROI to further characterize the directionality of these cluster‐level alterations.

Pearson correlations were adopted to explore the associations between significant neuroimaging indices (topological properties or FC) and clinical symptom scores. In addition, disease duration was included in correlation analyses as a variable of interest, given prior evidence suggesting its potential association with brain functional alterations in chronic pain conditions [25]. Mediation analyses were implemented utilizing the PROCESS macro in SPSS Statistics for variables with significant correlations, aiming to assess the significance of mediation effects. Point estimates, standard errors, and bias‐corrected 95% confidence intervals (CI) for each indirect effect were estimated via a bootstrap procedure containing 5000 random samples, which was used to obtain these key statistical parameters. Finally, ROC curve analysis [26] was employed to ascertain the discriminative ability of significant rs‐fMRI indices for dIBS versus ndIBS patients.

## 3. Results

### 3.1. Demographic and Clinical Characteristics

All enrolled subjects’ demographic and clinical traits are summarized in Table 2. No statistically significant intergroup differences were identified in sex (*p* = 0.456), age (*p* = 0.087), or education level (*p* = 0.164) and were thus matched on these demographic characteristics. The two patient groups exhibited no significant difference in disease duration (*p* = 0.331). As expected, both the dIBS and ndIBS groups exhibited higher HAMD‐17 scores and HAMA‐14 scores than HCs group (*p* < 0.001). Regarding gastrointestinal symptoms, the dIBS group presented significantly higher GSRS total scores compared to the ndIBS group (*p* < 0.001). Further subscale analysis revealed that the dIBS group exhibited significantly greater severity in abdominal pain, reflux, indigestion, and diarrhea subscores than the ndIBS group (all *p* < 0.05).

**Table 2:** Demographic and clinical characteristics of participants.

Variable	dIBS (*n* = 28)	ndIBS (*n* = 21)	HCs (*n* = 36)	*p*‐values
Sex (female/male)	11/17	9/12	10/26	0.456^a^
Age (years)	36.36 ± 7.31	32.29 ± 9.96	31.67 ± 8.85	0.087^b^
Education (years)	11.82 ± 3.09	12.95 ± 2.58	13.28 ± 3.32	0.164^b^
Duration of IBS (months)	20.64 ± 9.01	18.57 ± 4.00	–	0.331^c^
GSRS total score	42.11 ± 11.76	23.43 ± 5.42	–	<0.001^c^
GSRS abdominal pain	10.07 ± 4.43	4.52 ± 1.78	–	<0.001^c^
GSRS reflux	3.89 ± 1.59	2.71 ± 1.01	–	0.003^c^
GSRS indigestion	13.96 ± 3.98	5.52 ± 1.86	–	<0.001^c^
GSRS diarrhea	9.61 ± 4.31	6.52 ± 2.71	–	0.006^c^
GSRS constipation	4.57 ± 1.57	4.24 ± 0.77	–	0.377^c^
HAMD‐17 scores	12.75 ± 1.71 ^∗^	3.71 ± 1.38 ^∗∗^ ^∗∗∗^	0.42 ± 0.77	<0.001^b^
HAMA‐14 scores	8.68 ± 2.18 ^∗^	3.05 ± 1.77 ^∗∗^ ^∗∗∗^	0.56 ± 0.81	<0.001^b^

*Note:* Data are shown as mean ± standard deviation.

Abbreviations: dIBS, IBS patients with depressive symptoms; GSRS, gastrointestinal symptoms rating scale; HAMA‐14, Hamilton anxiety scale 14; HAMD‐17, Hamilton depressive scale 17; HCs, healthy controls; IBS, irritable bowel syndrome; ndIBS, IBS patients without depressive symptoms.

^a^ *χ* ^2^.

^b^ *p*‐values of one‐way ANOVA and post hoc comparisons (dIBS vs. HCs,  ^∗^ *p* < 0.001; ndIBS vs. HCs,  ^∗∗^ *p* < 0.001; dIBS vs. ndIBS,  ^∗∗∗^ *p* < 0.001).

^c^Unpaired *t* test.

### 3.2. Topological Property of Amygdala Subregions

**Figure 1:**
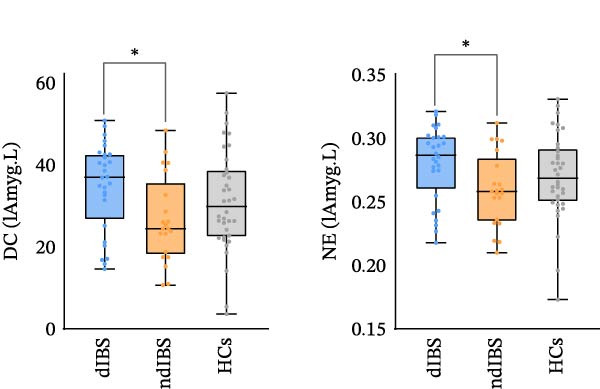
Differences in topological property of amygdala subregions among the three groups based on ANCOVA. Abbreviations: ANCOVA, analysis of covariance; DC, degree centrality; dIBS, IBS patients with depressive symptoms; HCs, healthy controls; IBS, irritable bowel syndrome; L, left; lAmyg, lateral amygdala; ndIBS, IBS patients without depressive symptoms; NE, nodal efficiency;  ^∗^ *p*  < 0.05.

Among all nodal network metrics across the four amygdala subregions, only the left lAmyg showed a significant group effect for DC (*p* = 0.033), while its NE did not reach conventional statistical significance (*p* = 0.050) but showed a trend toward a group effect. Post hoc *t*‐tests demonstrated that, relative to ndIBS patients, dIBS patients exhibited significantly increased DC (*p* = 0.015) and Ne (*p* = 0.025) in the left lAmyg (Figure 1).

### 3.3. FC of Amygdala Subregions

**Figure 2:**
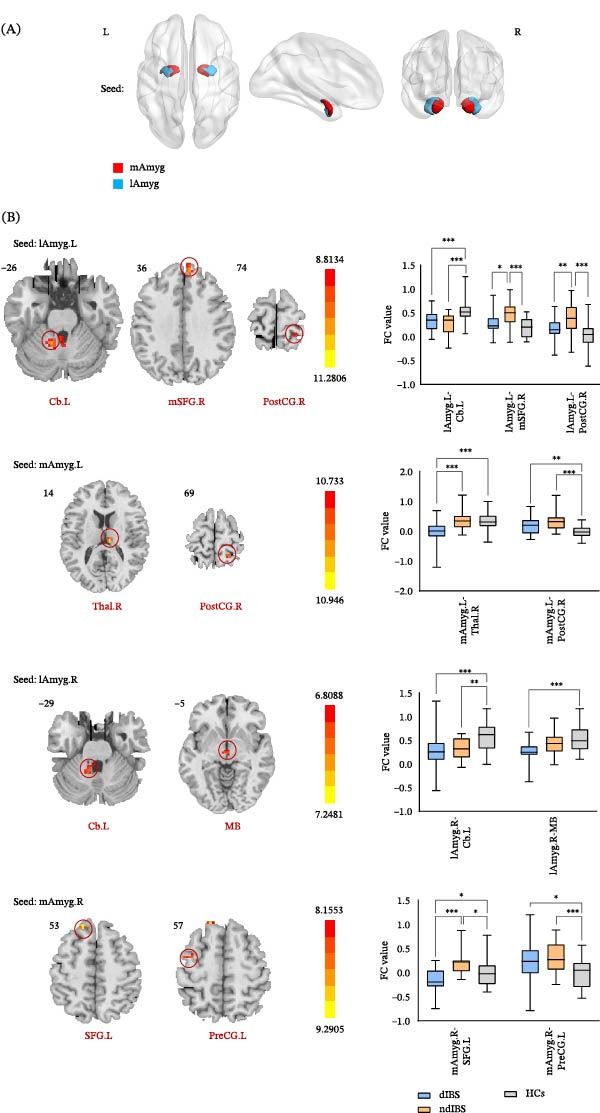
(A) The four subregions of the amygdala in the bilateral hemisphere. (B) ANCOVA (left) and post hoc *t* test (right) results of whole‐brain FC analysis using amygdala subregions as seed region. GRF corrected, cluster‐level: *p*  < 0.05, voxel‐level: *p*  < 0.005. Abbreviations: ANCOVA, analysis of covariance; Cb, cerebellum; dIBS, IBS patients with depressive symptoms; FC, functional connectivity; HCs, healthy controls; IBS, irritable bowel syndrome; L/R, left/right; lAmyg, lateral amygdala; mAmyg, medial amygdala; MB, midbrain; mSFG, medial superior frontal gyrus; ndIBS, IBS patients without depressive symptoms; PostCG, postcentral gyrus; preCG, precentral gyrus; SFG, superior frontal gyrus; Thal, thalamus;  ^∗^ *p*  < 0.05,  ^∗∗^ *p* < 0.01,  ^∗∗∗^ *p* < 0.001.

Significant group differences in FC were observed across all amygdala subregions (Table 3; Figure 2). These alterations primarily involved prefrontal, sensorimotor, and subcortical regions.

**Table 3:** The significant clusters of amygdala‐related FC differences in functional connectivity analysis among three groups.

Seed	Brain regions	MNI coordinates			Voxels	*F*/*T* value
		*x*	*y*	*z*		
One‐way ANCOVA results						
Left lAmyg	Left cerebellum	−12	−48	−27	65	12.150
	Right medial superior frontal gyrus	9	51	36	15	9.660
	Right postcentral gyrus	24	−45	75	17	11.497
Left mAmyg	Right thalamus	9	−21	15	18	12.306
	Right postcentral gyrus	24	−45	75	18	12.669
Right lAmyg	Left cerebellum	−9	−45	−30	28	9.047
	Left midbrain	0	−15	−6	10	9.738
Right mAmyg	Left superior frontal gyrus	−15	36	54	11	11.641
	Left precentral gyrus	−36	−3	60	10	7.515
dIBS vs. ndIBS						
Left lAmyg	Right medial superior frontal gyrus	9	51	36	15	−2.784
	Right postcentral gyrus	24	−45	75	17	−3.025
Left mAmyg	Right thalamus	9	−21	15	18	−3.962
Right mAmyg	Left superior frontal gyrus	−15	36	54	11	−4.823
dIBS vs. HCs						
Left lAmyg	Left cerebellum	−12	−48	−27	65	−3.759
Left mAmyg	Right thalamus	9	−21	15	18	−4.547
	Right postcentral gyrus	24	−45	75	18	3.284
Right lAmyg	Left cerebellum	−9	−45	−30	28	−3.849
	Left midbrain	0	−15	−6	10	−4.295
Right mAmyg	Left superior frontal gyrus	−15	36	54	11	−2.469
	Left precentral gyrus	−36	−3	60	10	2.553
ndIBS vs. HCs						
Left lAmyg	Left cerebellum	−12	−48	−27	65	−4.430
	Right medial superior frontal gyrus	9	51	36	15	4.392
	Right postcentral gyrus	24	−45	75	17	4.792
Left mAmyg	Right postcentral gyrus	24	−45	75	18	4.812
Right lAmyg	Left cerebellum	−9	−45	−30	28	−3.203
Right mAmyg	Left superior frontal gyrus	−15	36	54	11	2.806
	Left precentral gyrus	−36	−3	60	10	3.697

*Note:* GRF‐corrected, cluster‐level: *p*  < 0.05, voxel‐level: *p* < 0.005.

Abbreviations: ANCOVA, analysis of covariance; dIBS, IBS patients with depressive symptoms; HCs, healthy controls; IBS, irritable bowel syndrome; lAmyg, lateral amygdala; mAmyg, medial amygdala; MNI, Montreal Neurological Institute; ndIBS, IBS patients without depressive symptoms.

Compared with the ndIBS group, the dIBS group demonstrated decreased FC between the left lAmyg and the right medial SFG and postcentral gyrus, between the left mAmyg and the right thalamus, and between the right mAmyg and the left SFG (Table 3; Figure 2).

Compared with HCs, both IBS subgroups exhibited decreased FC between the bilateral lAmyg and the left cerebellum, along with increased FC between the left mAmyg and right postcentral gyrus, and between the right mAmyg and left precentral gyrus. Additionally, the dIBS group demonstrated reduced FC between the right lAmyg and the midbrain, between the left mAmyg and the right thalamus, and between the right mAmyg and the left SFG. The ndIBS group exhibited increased left lAmyg‐related FC in the right medial SFG and the right postcentral gyrus and increased right mAmyg‐related FC in the left SFG (Table 3; Figure 2).

### 3.4. Correlation Analysis

In all IBS patients, nodal metrics of the left lAmyg showed positively correlations with emotional symptoms. Specifically, both DC and Ne values were significantly associated with HAMD scores (*p* < 0.05).

Conversely, amygdala subregional FC showed consistent negative associations with both gastrointestinal and emotional symptoms. Higher GSRS, HAMD, and HAMA scores were correlated with reduced connectivity in the following pathways: the left lAmyg with the right postcentral gyrus, the left mAmyg with the right thalamus, and the right mAmyg with the left SFG (all *p* < 0.05).

**Figure 3:**
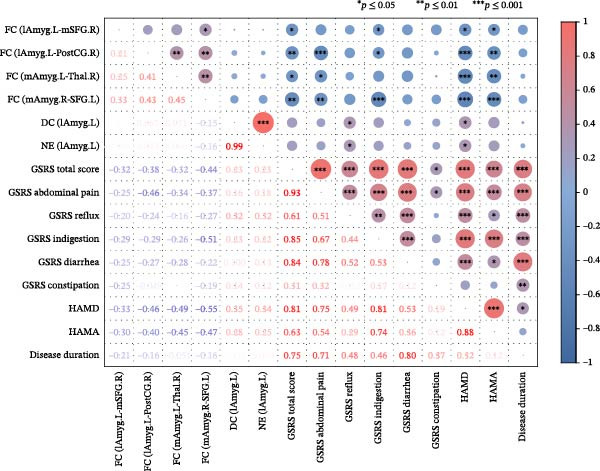
Correlation analysis among amygdala subregion‐related differential FC values, nodal topological properties, and clinical symptoms of IBS patients. Abbreviations: DC, degree centrality; FC, functional connectivity; GSRS, gastrointestinal symptoms rating scale; HAMA, Hamilton anxiety scale; HAMD, Hamilton depressive scale; IBS, irritable bowel syndrome; mSFG, medial superior frontal gyrus; NE, nodal efficiency; PostCG, postcentral gyrus; SFG, superior frontal gyrus; Thal, thalamus.

Further analyses of GSRS subscales revealed that associations for abdominal pain and dyspepsia scores mirrored the patterns observed for the total GSRS score (Supporting Information). No significant correlations were found between neuroimaging indices and disease duration. These correlation results are presented in Figure 3. To further evaluate the robustness of these clinical correlations, the false discovery rate (FDR) method was additionally applied. A complete overview of the statistical metrics, including both the uncorrected *p*‐values and FDR‐corrected *q*‐values for all comparisons, is provided in Table S1. Notably, 11 core correlations remained statistically significant after FDR correction (*q* < 0.05), primarily involving the FC of amygdala subregions with the right postcentral gyrus, right thalamus, and left SFG in relation to GSRS, HAMD, and HAMA scores (visualized in Figure S1).

### 3.5. Mediation Analysis

**Figure 4:**
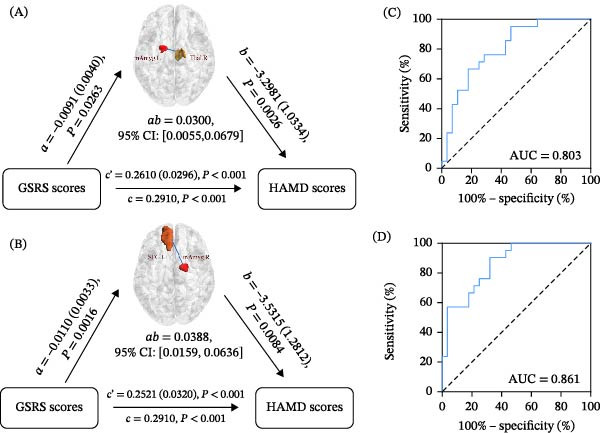
(A,B) Results of the mediated analysis between amygdala subregion‐related FC values and clinical symptoms in IBS patients. (C,D) Receiver operating characteristic (ROC) curve and the area under the curve (AUC) that left mAmyg‐right thalamus and right mAmyg‐left SFG FC value could be used to differentiate between dIBS and ndIBS patients. Abbreviations: FC, functional connectivity; GSRS, gastrointestinal symptoms rating scale; HAMD, Hamilton depression rating scale; IBS, irritable bowel syndrome; L/R, left/right; lAmyg, lateral amygdala; mAmyg, medial amygdala; SFG, superior frontal gyrus; Thal, thalamus.

Mediation analysis identified two distinct FC alterations that partially accounted for the link between gastrointestinal symptoms and depressive manifestations (Figure 4).

For left mAmyg‐right thalamus FC, GSRS scores were negatively associated with this connectivity (path a: *a* = −0.0091, *p* = 0.0263), which was further negatively related to HAMD scores (path b: *b* = −3.2981, *p* = 0.0026). The direct association between GSRS and HAMD remained significant after accounting for this mediator (path c’: *c*’ = 0.2610, *p*  < 0.001), with a significant indirect effect (*ab* = 0.0300, 95% CI [0.0055, 0.0679]) verified by bootstrap analysis.

For right mAmyg‐left SFG FC, GSRS scores correlated negatively with the connectivity (path a: *a* = −0.0110, *p* = 0.0016), which was negatively associated with HAMD scores (path b: *b* = −3.5315, *p* = 0.0084). The direct effect of GSRS on depressive symptoms remained significant after mediator adjustment (path c′: *c*′ = 0.2521, *p*  < 0.001), and bootstrap analysis confirmed a significant indirect effect (*ab* = 0.0388, 95% CI [0.0159, 0.0636]).

### 3.6. ROC Curve Analysis

The aberrant FC values demonstrated high discriminative power between dIBS and ndIBS patients; the aberrant FC value between the right mAmyg and left SFG exhibited an area under the curve (AUC) of 0.861 (*p* < 0.001; 95% CI: [0.761, 0.961]), whereas that between the left mAmyg and right thalamus presented an AUC of 0.803 (*p* < 0.001; 95% CI: [0.681, 0.925]) (Figure 4).

Furthermore, additional ROC analyses were performed between HCs and the two IBS subgroups to explore the diagnostic specificity of these markers. Specifically, the FC between the left mAmyg and right thalamus appeared to differentiate dIBS from HCs, whereas ndIBS was primarily associated with altered connectivity between the left amygdala and sensorimotor regions. The relevant data and ROC curves for these comparisons are available in Table S2 and Figure S2.

## 4. Discussion

By integrating graph theory analysis and seed‐based FC analyses, this study reveals three principal findings: (1) increased centrality of the left lAmyg in dIBS, (2) disrupted amygdala subregion‐specific connectivity involving prefrontal, midbrain, and sensorimotor systems, and (3) these alterations are associated with both gastrointestinal and affective symptoms. Importantly, mediation and ROC analyses further suggest that these subregion‐specific circuits may serve as candidate biomarkers for the somatic–affective interactions characterizing dIBS.

### 4.1. Elevated Centrality of the Left lAmyg in dIBS

DC quantifies the total number of functional connections a node establishes across the entire brain, while Ne reflects its efficiency in information transmission [27]. Elevated DC and Ne indicate that the lAmyg functions as an overactive functional hub in dIBS. This finding aligns with the concept of sustained vigilance in IBS, whereby heightened amygdala activity facilitates enhanced perception and negative appraisal of visceral signals [28]. As a core subregion involved in salience detection and initial threat appraisal, the lAmyg integrates sensory inputs from the thalamus, sensory cortex, and brainstem [29]. Its elevated centrality may reflect amplified coupling between sensory and affective systems, promoting reciprocal reinforcement between gastrointestinal symptoms and negative emotions. Consistent with this interpretation, chronic stress induces lAmyg hyperactivity and visceral hypersensitivity in animal models, whereas inhibition of this activity mitigates anxiety‐ and depression‐like behaviors [30, 31]. Notably, these topological changes were specific to dIBS, suggesting that lAmyg‐centered network hyperactivity may underlie somatic–affective comorbidity rather than the gastrointestinal pathology alone. From a clinical perspective, the lAmyg as a potential network‐level target for interventions aimed at reducing hypervigilance and symptom amplification.

### 4.2. Dysregulated Amygdala Subregion‐Related Connectivity in dIBS

#### 4.2.1. Amygdala Subregion‐Prefrontal Disconnection

The SFG modulates negative emotions through top–down suppression of amygdala hyperactivity, with its medial and dorsal subregions critical for cognitive control and emotion regulation [32]. Aberrant resting‐state FC within this amygdala‐prefrontal circuit correlates with clinical pain duration and emotional status in IBS. Insufficient prefrontal inhibitory control may amplify negative emotions arising from visceral sensations, contributing to a reciprocal interaction between gastrointestinal symptoms and affective disturbance [28, 33]. A similar pattern has been observed in chronic pain comorbid with depression, where decreased amygdala‐medial SFG connectivity is linked to greater symptom severity [10]. At the subregional level, the lAmyg is primarily involved in salience detection, whereas the mAmyg contributes to affective valuation, both under prefrontal modulation [15]. Disruption of these pathways has been implicated in depression and somatic symptom amplification [34–36]. Extending these observations, diminished FC within the amygdala subregion‐prefrontal circuits in dIBS may reflect impaired top–down regulation of visceral‐affective processing. The observed associations with gastrointestinal symptoms, particularly abdominal pain and reflux, further suggest that this pathway is more closely related to symptom‐specific dimensions rather than global symptom burden [22]. Collectively, these findings support a role of prefrontal‐amygdala dysregulation in the interaction between visceral perception and emotional processing in dIBS. The weakened top–down control may exacerbate the mutual reinforcement of negative emotions and visceral sensations, which may a core neurobiological for the somatic–emotional comorbidity in dIBS patients.

#### 4.2.2. lAmyg‐Midbrain Autonomic Network Connectivity Deficits

The midbrain, particularly the periaqueductal gray (PAG), is a core component of the central autonomic network (CAN) and regulates stress responses and autonomic functions [33]. The PAG interacts with the amygdala, with the lAmyg processing aversive visceral signals and conveying this information to autonomic control systems [37]. Dysfunction of the PAG‐amygdala circuit disrupts aversive processing and affects downstream emotional and cognitive networks [38]. In IBS, CAN dysregulation and altered brainstem and amygdala activity related to norepinephrine signaling have been linked to symptom severity [39]. Within this context, reduced lAmyg‐midbrain connectivity in dIBS may indicate a specific deficit in integrating autonomic‐emotional responses to stress and visceral signals, which occurs against the broader backdrop of CAN upregulation [33]. Clinically, this highlights the relevance of brainstem‐limbic circuits as potential targets for improving stress adaptation and autonomic regulation.

#### 4.2.3. Disrupted Amygdala Subregion‐SMN Integration

The sensorimotor network (SMN), including the thalamus, postcentral gyrus, and precentral gyrus, is essential for processing and responding to visceral sensory input. Within this network, the thalamus acts as a primary relay that forwards sensory signals to the amygdala for emotional evaluation [40]. Prior studies have demonstrated abnormal thalamus‐amygdala connectivity in IBS and chronic pain [33, 41, 42]. Consistent with these findings, we observed that amygdala subregion‐SMN connectivity is associated with abdominal pain and dyspepsia subscores, indicating a role in the sensory‐discriminative dimension of visceral pain [22]. This is further supported by our ROC analyses, where the mAmyg‐thalamus circuit specifically distinguished dIBS from both HCs and ndIBS patients, suggesting its role as a sensitive biomarker for the affective dimensions of the disorder.

The involvement of other SMN components provides further evidence for disrupted visceral processing. Specifically, altered connectivity with the postcentral gyrus and precentral gyrus likely reflects impaired visceral sensory encoding and sensorimotor integration [43–45]. These region‐specific patterns link amygdala‐SMN circuits directly to abdominal pain and dyspepsia scores, indicating their contribution to the sensory‐discriminative dimension of pain. Notably, while the thalamic pathway was central to dIBS, the discriminative power for ndIBS was primarily driven by these sensorimotor regions and the cerebellum. Cerebellar–amygdala interactions, potentially mediated via thalamic pathways, are thought to contribute to pain anticipation [46–49]. Together, these subregion‐specific integration deficits underlie the abnormal visceral perception in dIBS and suggest that targeting mAmyg‐sensorimotor pathways may improve both symptom perception and emotional regulation.

Several inherent limitations of this investigation warrant acknowledgment. First, the relatively modest sample size may limit the generalizability of the findings. This was partly due to the use of a previously established cohort with strict inclusion and exclusion criteria, which constrained further expansion. Future studies with larger samples are warranted to validate and extend the present findings. Second, although mediation analysis statistically suggests a potential causal relationship, longitudinal studies are required to confirm whether the observed amygdala connectivity alterations precede or result from symptom comorbidity. Finally, although the current study lacks a depression only control group, our findings align with an emerging transdiagnostic framework in gut–brain research. Recent cross‐disorder investigations highlight the value of identifying shared and distinct biomarkers across related conditions [22]. Future studies utilizing similar cross‐condition designs will help clarify the unique neurobiological contributions of IBS and depression.

## 5. Conclusion

lAmyg hyperactivity correlates with sustained vigilance to visceral signals and amplified negative emotional valence in dIBS. Dysregulated connectivity between amygdala subregions and the PFC, sensorimotor, and autonomic networks may reflect disrupted visceral‐affective integration. Notably, decreased FC between the mAmyg and sensorimotor regions may serve as a diagnostic biomarker for dIBS. These findings highlight the amygdala subregion‐centered network as a promising target for individualized neuromodulation or psychological interventions aimed at simultaneously alleviating gastrointestinal and affective symptoms.

## Author Contributions

Yidan Liang contributed to manuscript writing. Rongting Hou, Liqiang Wu, Yihan Jin, Ruoyu Tang, Yun Guo, and Xiaofei Chen contributed to data sources and data acquisition. Jie Li administered the project and contributed to the review and editing of the manuscript.

## Funding

This study was supported by the National Natural Science Foundation of China (Grant 82402216); the Zhejiang Provincial Natural Science Foundation of China (Grant LTGY24H180016); the Natural Science Foundation of Hangzhou (Grant 2025SZRJJ0059); and the Medical Science and Technology Project of Zhejiang Province (Grants 2024KY198 and 2025KY139).

## Disclosure

All authors reviewed and approved the final version of the manuscript.

## Ethics Statement

The study was conducted in accordance with the Declaration of Helsinki and approved by the Ethics Committee of the Affiliated Hospital of Hangzhou Normal University.

## Conflicts of Interest

The authors declare no conflicts of interest.

## Supporting Information

Additional supporting information can be found online in the Supporting Information section.

## Supporting information


**Supporting Information 1** The Supporting Information includes the detailed results of the clinical subscale correlation analyses and additional diagnostic specificity evaluations. This encompasses the comprehensive statistical metrics for clinical symptom correlations (Table S1, Figure S1) as well as the ROC curve parameters and visualizations comparing HCs and IBS subgroups (Table S2, Figure S2). Table S1: FDR‐corrected correlations among amygdala subregion‐related FC alterations, nodal topological properties, and clinical symptoms in IBS patients. Figure S1: Heatmap of FDR‐corrected correlations among amygdala subregion‐related differential FC values, nodal topological properties, and clinical symptoms in IBS patients. Table S2: ROC analysis and AUC values for amygdala subregion‐related functional connectivity alterations distinguishing IBS subgroups from HCs. Figure S2: Receiver operating characteristic (ROC) curves of amygdala subregion‐related functional connectivity alterations distinguishing IBS subgroups from HCs.

## Data Availability

The data that support the findings of this study are available from the corresponding authors upon request.
