# Supplementary material for: Aberrant Nodal Topological Properties and Functional Connectivity of Amygdala Subregions Underlie Emotion–Visceral Integration Impairment in IBS With Depressive Symptoms
Source: Neural Plast. 2026 Jul 20;2026:7203336. doi: 10.1155/np/7203336 (PMC13382785; doi:10.1155/np/7203336)
Supplement: Supplementary file 1 — Supporting Information 1 The Supporting Information includes the detailed results of the clinical subscale correlation analyses and additional diagnostic specificity evaluations. This encompasses the comprehensive statistical metrics for clinical symptom correlations (Table S1, Figure S1) as well as the ROC curve parameters and visualizations comparing HCs and IBS subgroups (Table S2, Figure S2). Table S1: FDR‐corrected correlations among amygdala subregion‐related FC alterations, nodal topological properties, and clinical symptoms in IBS patients. Figure S1: Heatmap of FDR‐corrected correlations among amygdala subregion‐related differential FC values, nodal topological properties, and clinical symptoms in IBS patients. Table S2: ROC analysis and AUC values for amygdala subregion‐related functional connectivity alterations distinguishing IBS subgroups from HCs. Figure S2: Receiver operating characteristic (ROC) curves of amygdala subregion‐related functional connectivity alterations distinguishing IBS subgroups from HCs. [file NP-2026-7203336-s001.docx]

**Supplementary material**

**Title:**

**Aberrant nodal topological properties and functional connectivity of amygdala subregions underlie emotion-visceral integration impairment in IBS with depressive symptoms**

**Authors:**

Yidan Liang^1,2^, Rongting Hou^1,2^, Liqiang Wu^1,2^, Yihan Jin^1,2^, Ruoyu Tang^1^, Yun Guo^3^, Xiaofei Chen^2^, Jie Li^1,2,4*^

**Affiliation/address:**

^1^ School of Clinical Medicine, The Affiliated Hospital of Hangzhou Normal University, Hangzhou Normal University, Hangzhou, China
^2^ Department of Radiology, The Affiliated Hospital of Hangzhou Normal University, Hangzhou, China
^3^ Department of Gastroenterology, The Affiliated Hospital of Hangzhou Normal University, Hangzhou, China

^4^ Centre for Cognition and Brain Disorders, The Affiliated Hospital of Hangzhou Normal University, Hangzhou, China

**Corresponding authors:**

Jie Li
Address: No.126, Wenzhou Street, Gongshu District, Hangzhou, Zhejiang 310015, China.

E-mail: jie_sweethz@163.com

**Supplementary Table S1.** **FDR-corrected correlations among amygdala subregion-related FC alterations, nodal topological properties, and clinical symptoms in IBS patients**

|  | **Brain region** | **Clinical variable** | **r** | ***P* (uncorrected)** | ***q* (FDR)** |
| --- | --- | --- | --- | --- | --- |
| 1 | FC(lAmyg.L - mSFG.R) | GSRS total score | -0.32 | 0.024* | 0.075 |
| 2 | FC(lAmyg.L - mSFG.R) | GSRS abdominal pain | -0.25 | 0.078 | 0.146 |
| 3 | FC(lAmyg.L - mSFG.R) | GSRS reflux | -0.20 | 0.173 | 0.240 |
| 4 | FC(lAmyg.L - mSFG.R) | GSRS indigestion | -0.29 | 0.045* | 0.111 |
| 5 | FC(lAmyg.L - mSFG.R) | GSRS diarrhoea | -0.25 | 0.086 | 0.149 |
| 6 | FC(lAmyg.L - mSFG.R) | GSRS constipation | -0.25 | 0.082 | 0.148 |
| 7 | FC(lAmyg.L - mSFG.R) | HAMD | -0.33 | 0.020* | 0.071 |
| 8 | FC(lAmyg.L - mSFG.R) | HAMA | -0.30 | 0.035* | 0.094 |
| 9 | FC(lAmyg.L - mSFG.R) | Disease duration | -0.21 | 0.139 | 0.197 |
| 10 | FC(lAmyg.L - PostCG.R) | GSRS total score | -0.38 | 0.007** | 0.039* |
| 11 | FC(lAmyg.L - PostCG.R) | GSRS abdominal pain | -0.46 | 0.001** | 0.008** |
| 12 | FC(lAmyg.L - PostCG.R) | GSRS reflux | -0.24 | 0.101 | 0.171 |
| 13 | FC(lAmyg.L - PostCG.R) | GSRS indigestion | -0.29 | 0.041* | 0.106 |
| 14 | FC(lAmyg.L - PostCG.R) | GSRS diarrhoea | -0.27 | 0.058 | 0.121 |
| 15 | FC(lAmyg.L - PostCG.R) | GSRS constipation | -0.05 | 0.741 | 0.769 |
| 16 | FC(lAmyg.L - PostCG.R) | HAMD | -0.46 | 0.001** | 0.008** |
| 17 | FC(lAmyg.L - PostCG.R) | HAMA | -0.40 | 0.004** | 0.027* |
| 18 | FC(lAmyg.L - PostCG.R) | Disease duration | -0.16 | 0.271 | 0.336 |
| 19 | FC(mAmyg.L - Thal.R) | GSRS total score | -0.32 | 0.026* | 0.075 |
| 20 | FC(mAmyg.L - Thal.R) | GSRS abdominal pain | -0.34 | 0.017* | 0.070 |
| 21 | FC(mAmyg.L - Thal.R) | GSRS reflux | -0.16 | 0.280 | 0.336 |
| 22 | FC(mAmyg.L - Thal.R) | GSRS indigestion | -0.26 | 0.069 | 0.138 |
| 23 | FC(mAmyg.L - Thal.R) | GSRS diarrhoea | -0.28 | 0.050 | 0.117 |
| 24 | FC(mAmyg.L - Thal.R) | GSRS constipation | 0.02 | 0.910 | 0.910 |
| 25 | FC(mAmyg.L - Thal.R) | HAMD | -0.49 | 0.000*** | 0.006** |
| 26 | FC(mAmyg.L - Thal.R) | HAMA | -0.45 | 0.001** | 0.009** |
| 27 | FC(mAmyg.L - Thal.R) | Disease duration | -0.05 | 0.727 | 0.769 |
| 28 | FC(mAmyg.R - SFG.L) | GSRS total score | -0.44 | 0.002** | 0.010* |
| 29 | FC(mAmyg.R - SFG.L) | GSRS abdominal pain | -0.37 | 0.009** | 0.045* |
| 30 | FC(mAmyg.R - SFG.L) | GSRS reflux | -0.27 | 0.056 | 0.121 |
| 31 | FC(mAmyg.R - SFG.L) | GSRS indigestion | -0.51 | 0.000*** | 0.005** |
| 32 | FC(mAmyg.R - SFG.L) | GSRS diarrhoea | -0.22 | 0.131 | 0.191 |
| 33 | FC(mAmyg.R - SFG.L) | GSRS constipation | -0.19 | 0.190 | 0.257 |
| 34 | FC(mAmyg.R - SFG.L) | HAMD | -0.55 | 0.000*** | 0.002** |
| 35 | FC(mAmyg.R - SFG.L) | HAMA | -0.47 | 0.001** | 0.008** |
| 36 | FC(mAmyg.R - SFG.L) | Disease duration | -0.16 | 0.278 | 0.336 |
| 37 | DC(lAmyg_L) | GSRS total score | 0.23 | 0.119 | 0.183 |
| 38 | DC(lAmyg_L) | GSRS abdominal pain | 0.16 | 0.261 | 0.336 |
| 39 | DC(lAmyg_L) | GSRS reflux | 0.32 | 0.024* | 0.075 |
| 40 | DC(lAmyg_L) | GSRS indigestion | 0.23 | 0.104 | 0.171 |
| 41 | DC(lAmyg_L) | GSRS diarrhoea | 0.10 | 0.496 | 0.546 |
| 42 | DC(lAmyg_L) | GSRS constipation | 0.14 | 0.328 | 0.385 |
| 43 | DC(lAmyg_L) | HAMD | 0.35 | 0.013* | 0.060 |
| 44 | DC(lAmyg_L) | HAMA | 0.28 | 0.052 | 0.117 |
| 45 | DC(lAmyg_L) | Disease duration | 0.03 | 0.859 | 0.876 |
| 46 | NE(lAmyg_L) | GSRS total score | 0.23 | 0.109 | 0.173 |
| 47 | NE(lAmyg_L) | GSRS abdominal pain | 0.18 | 0.220 | 0.290 |
| 48 | NE(lAmyg_L) | GSRS reflux | 0.32 | 0.025* | 0.075 |
| 49 | NE(lAmyg_L) | GSRS indigestion | 0.22 | 0.123 | 0.185 |
| 50 | NE(lAmyg_L) | GSRS diarrhoea | 0.13 | 0.383 | 0.440 |
| 51 | NE(lAmyg_L) | GSRS constipation | 0.12 | 0.400 | 0.450 |
| 52 | NE(lAmyg_L) | HAMD | 0.34 | 0.018* | 0.070 |
| 53 | NE(lAmyg_L) | HAMA | 0.25 | 0.078 | 0.146 |
| 54 | NE(lAmyg_L) | Disease duration | 0.05 | 0.736 | 0.769 |

Abbreviations: FC, functional connectivity; DC, degree centrality; NE, nodal efficiency; lAmyg, lateral amygdala; mAmyg, medial amygdala; L/R, left/right; mSFG, medial superior frontal gyrus; PostCG, postcentral gyrus; Thal, thalamus; SFG, superior frontal gyrus; GSRS, gastrointestinal symptoms rating scale; HAMD, Hamilton depressive scale; HAMA, Hamilton anxiety scale. Note: *P* (uncorrected), uncorrected *P*-value; *q* (FDR), FDR-corrected *q*-value. (* *P* ≤ 0.05, ***P* ≤ 0.01, ****P* ≤ 0.001)


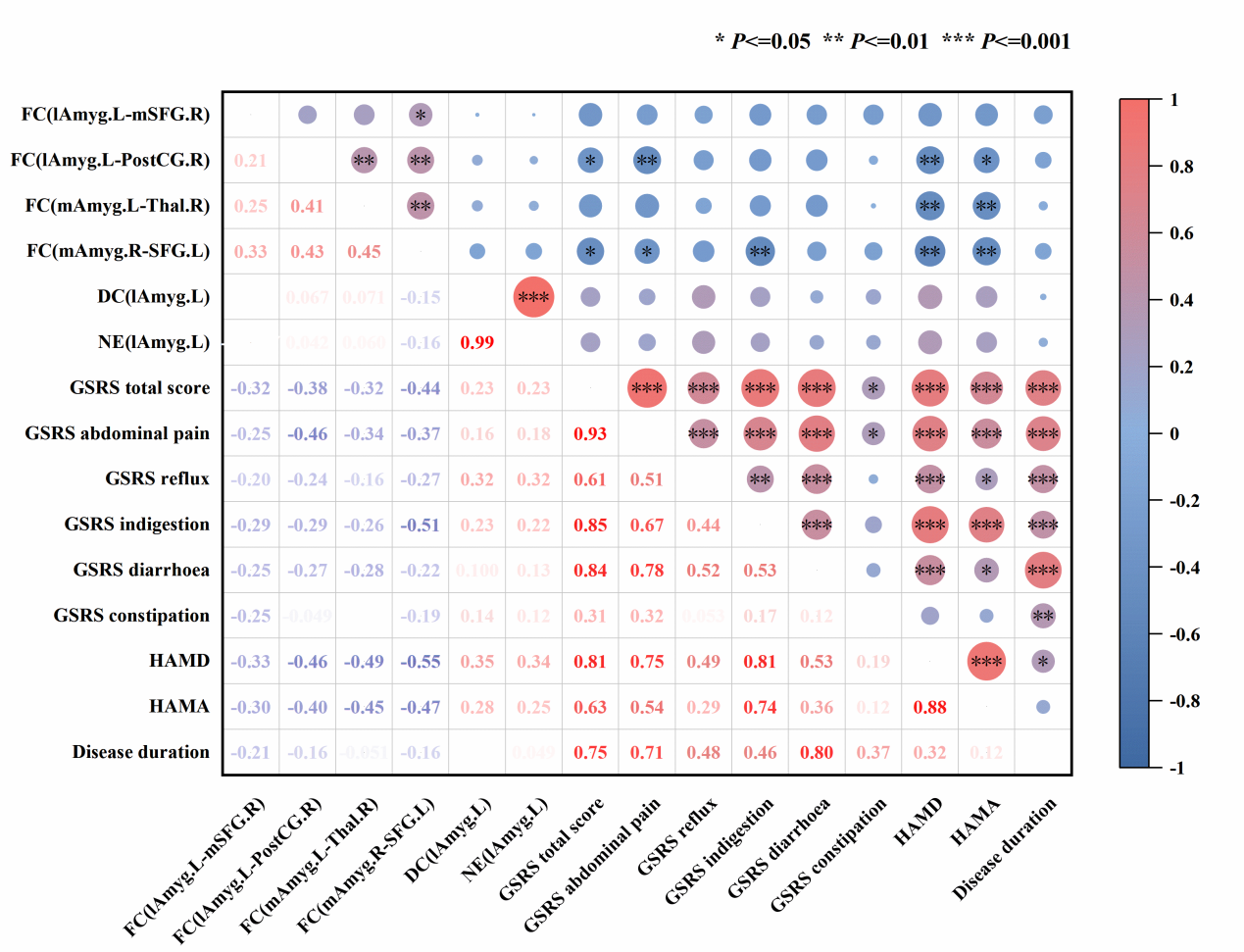


**Supplementary Fig. S1. Heatmap of FDR-corrected correlations among amygdala subregion-related differential FC values, nodal topological properties, and clinical symptoms in IBS patients.** Abbreviations: FC, functional connectivity; DC, degree centrality; NE, nodal efficiency; lAmyg, lateral amygdala; mAmyg, medial amygdala; L/R, left/right; mSFG, medial superior frontal gyrus; PostCG, postcentral gyrus; Thal, thalamus; SFG, superior frontal gyrus; GSRS, gastrointestinal symptoms rating scale; HAMD, Hamilton depressive scale; HAMA, Hamilton anxiety scale.

**Supplementary Table S2. ROC analysis and AUC values for amygdala subregion-related functional connectivity alterations distinguishing IBS subgroups from healthy controls**

| **Comparison** | **Functional connectivity feature** | **AUC** | **95% CI** | ***P* value** |
| --- | --- | --- | --- | --- |
| dIBS vs. HCs | Left mAmyg – right thalamus | 0.802 | 0.692–0.911 | <0.001 |
| ndIBS vs. HCs | Left lAmyg – left cerebellum | 0.812 | 0.701–0.924 | <0.001 |
| ndIBS vs. HCs | Left lAmyg – right postcentral gyrus | 0.812 | 0.681–0.943 | <0.001 |
| ndIBS vs. HCs | Left mAmyg – right postcentral gyrus | 0.853 | 0.749–0.956 | <0.001 |

Abbreviations: IBS, irritable bowel syndrome; dIBS, IBS patients with depressive symptoms; ndIBS, IBS patients without depressive symptoms; HCs, healthy controls.

**
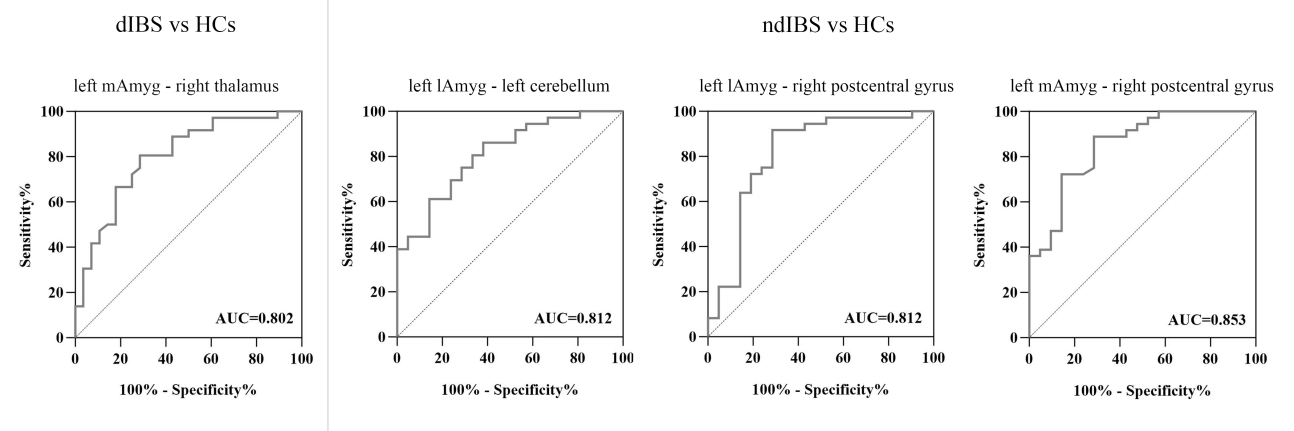
Supplementary Fig. S2. Receiver operating characteristic (ROC) curves of amygdala subregion-related functional connectivity alterations distinguishing IBS subgroups from healthy controls.** Abbreviations: IBS, irritable bowel syndrome; dIBS, IBS patients with depressive symptoms; ndIBS, IBS patients without depressive symptoms; HCs, healthy controls.
